# A methylation‐based mRNA signature predicts survival in patients with gastric cancer

**DOI:** 10.1186/s12935-020-01374-w

**Published:** 2020-07-06

**Authors:** Yang Li, Rongrong Sun, Youwei Zhang, Yuan Yuan, Yufeng Miao

**Affiliations:** 1 Department of Central Laboratory, Xuzhou Central Hospital, Xuzhou Clinical School of Xuzhou Medical University, Xuzhou, 221009 China; 2 Department of Medical Oncology, Xuzhou Central Hospital, Clinical School of Xuzhou Medical University, Xuzhou, 221009 China; 3 Department of Medical Oncology, The First Peoples’ Hospital of Wenling City, Wenling, 317500 China

**Keywords:** DNA methylation, MDEGs, Signature, Prognosis

## Abstract

**Background:**

Evidence suggests that altered DNA methylation plays a causative role in the occurrence, progression and prognosis of gastric cancer (GC). Thus, methylated-differentially expressed genes (MDEGs) could potentially serve as biomarkers and therapeutic targets in GC.

**Methods:**

Four genomics profiling datasets were used to identify MDEGs. Gene Ontology enrichment and Kyoto Encyclopaedia of Genes and Genomes pathway enrichment analysis were used to explore the biological roles of MDEGs in GC. Univariate Cox and LASSO analysis were used to identify survival-related MDEGs and to construct a MDEGs-based signature. The prognostic performance was evaluated in two independent cohorts.

**Results:**

We identified a total of 255 MDEGs, including 192 hypermethylation-low expression and 63 Hypomethylation-high expression genes. The univariate Cox regression analysis showed that 83 MDEGs were associated with overall survival. Further we constructed an eight-MDEGs signature that was independent predictive of prognosis in the training cohort. By applying the eight-MDEGs signature, patients in the training cohort could be categorized into high-risk or low-risk subgroup with significantly different overall survival (HR = 2.62, 95% CI 1.71–4.02, P < 0.0001). The prognostic value of the eight-MDEGs signature was confirmed in another independent GEO cohort (HR = 1.35, 95% CI 1.03–1.78, P = 0.0302) and TCGA-GC cohort (HR = 1.85, 95% CI 1.16–2.94, P = 0.0084). Multivariate cox regression analysis proved the eight-MDEGs signature was an independent prognostic factor for GC.

**Conclusion:**

We have thus established an innovative eight-MDEGs signature that is predictive of overall survival and could be a potentially useful guide for personalized treatment of GC patients.

## Background

Gastric cancer (GC) is the fourth most common cancer and the second leading cause of cancer-related mortality in the world [1, 2]. Surgery is the only curative treatment strategy in early GC, and conventional chemotherapy has displayed limited efficacy. Since a majority of patients are diagnosed with GC in locally advanced stage. Advanced disease carries a poor prognosis, with 5-year OS of 5–20% [3, 4]. Thus despite decreasing incidence, the mortality rate associated with GC remains relatively high. Therefore, new valid and reliable prognostic and predictive biomarkers for GC are needed to improve risk prediction and offer better information for guiding personalized therapy.

An increasing number of recent studies suggest that, in addition to genetic alterations, epigenetic alterations, including post-translational modifications of histones, noncoding RNAs, microRNAs, nucleosome positioning and DNA methylation of CpG islands are also involved in the initiation and progression of GC [5, 6]. By regulating and controlling the expression of cancer‐related genes, abnormal DNA methylation can seriously affect the occurrence and development of cancer [7, 8]. Studies have shown that DNA methylation which could provide biological markers for early diagnosis of cancer usually occurs in early cancer. Recently, a number of hyper-methylated tumor-suppressor genes and hypo-methylated tumor-promoting genes have also been found in GC, which were associated with oncogene positive transcriptional regulation in a multiple cellular processes. But to the best of our knowledge, there are no prior studies examining methylated-differentially expressed genes (MDEGs) on a genome-wide scale and focusing on predicting prognosis in GC. In the present study, we comprehensively analyzed Multi-Omics cohorts from the Gene Expression Omnibus (GEO) and TCGA to build a novel MDEGs-based signature that is predictive of prognosis and could potentially guide personalized therapy for GC patients.

## Material and method

### Data processing

All datasets and clinical information, as described in Table 1 and Additional file 1: Table S1, were downloaded from the GEO (https://www.ncbi.nlm.nih.gov/geo/) and TCGA (https://cancergenome.nih.gov/). Gene expression profiling of the GSE13911 [9] and GSE79973 [10] datasets was conducted using the GPL570 platform (Affymetrix Human Genome U133 plus 2.0 Array). The GSE13911 series included 38 GC and 31 normal gastric samples. And the GSE79973 series consisted of 10 paired GC and non-tumor samples. Gene methylation profiling of the GSE30601 [11] and GSE25869 [12] datasets was conducted using the GPL8490 platform (Illumina Human Methylation27 BeadChip), which included 27,578 highly informative CpG sites and more than 14,476 genes. The GSE30601 series consisted of 203 GC and 94 non-tumor samples. And the GSE25869 series consisted of 32 paired GC and non-tumor samples. The GSE15459 [13] series, including 192 GC samples with gene expression and clinical information, was used to extract a MDEGs-based prognostic signature. Two independent datasets collected from TCGA and GEO were used to test the prognostic ability of the MDEGs-based signature. For TCGA data, the normalised count values of level 3 gene expression data derived from Illumina HiSeqV2 were extracted as gene expression measurements. For data generated by the Affymetrix platforms, the Robust Multi-array Average algorithm [14] was used for preprocessing the raw data. For a data set generated by the Illumina microarray platform, the originally processed data were used. All gene expression measurements were log2 transformed. The Entrez IDs were used to map genes across microarray platforms.

**Table 1:** Datasets analyzed in this study

	Methylation dataset		Expression dataset		Training dataset	Validation dataset	
	GSE30601	GSE25869	GSE13911	GSE79973	GSE15459	TCGA-GC	GSE84437
Normal	94	32	31	10	–	–	–
Tumor	203	32	38	10	192	368	433
Platform	Illumina HM27		Affymetrix U133 Plus 2		Affymetrix U133 Plus 2	Illumina HiSeqV2	Illumina HT-12 V3

### Identification of MDEGs and functional enrichment analysis

We respectively used GEO2R and *T* test to screen for differentially methylated genes (DMGs) and differentially expressed genes (DEGs) between tumor and non-tumor samples. The P-values were adjusted using the Benjamini–Hochberg procedure for multiple testing to control the false discovery rate (FDR). Values of FDR < 0.05 were considered significant. The concordance score was calculated by binomial test. Further we correlated the level of RNA expression with the degree of DNA methylation to identify MDEGs. Hypomethylation-high expression genes were detected by overlapping hypo-methylated and up-regulated genes. Similarly, hypermethylation-low expression genes were detected by overlapping hyper-methylated and down-regulated genes.

Functional annotations in MDEGs were preformed using The Database for Annotation, Visualization and Integrated Discovery (DAVID, https://david.ncifcrf.gov/), which enriched gene oncology and pathways. Gene oncology involved three categories: biological processes, molecular function and cellular components. Pathway enrichment was carried out using the Kyoto Encyclopedia of Genes and Genomes (KEGG, https://www.kegg.jp/), and it contains information about genomes, chemical substances, biological pathways and diseases. The criterion for significant enrichment was P = 0.05.

### Establishment of MDEGs-based prognostic signature

The univariate Cox regression analysis was firstly performed based on MDEGs to calculate the association between the expression level of each gene and patient’s overall survival (OS) in training cohort. Those genes with P-values less than 0.05 were identified as prognosis-related MDEGs. Then, the prognosis-related MDEGs were further screened and confirmed by the Lasso regression. The basic idea of Lasso is to select the variables of the sample data under the constraint that the sum of the absolute values of the regression coefficients is less than a constant, so as to minimize the sum of the squares of the residuals and make some regression coefficients strictly equal to 0. To achieve the purpose of feature selection and obtain an optimal model subsequently, the variable with coefficient equal to 0 is regarded as a non-significant variable and is directly discarded. Using the combination of weighted MDEGs expression values, a risk scoring model was established and the risk scores were calculated as shown in the following equation: Risk score = expression of Gene 1 * β1 + expression of Gene 2 * β2 +···expression of Gene n * βn. βi is the regression coefficient of Gene i, which represents the contribution of Gene i to the prognostic risk score. Using the median risk score as the cutoff point, patients in each dataset were divided into low-risk or high-risk group correspondingly.

### Statistical analysis

The multivariate Cox proportional-hazards regression model was used to evaluate independent associations between prognostic signature and patient survival after adjusting for stage, age and gender. Hazard ratios (HRs) and 95% confidence intervals (CIs) were computed based on the Cox regression analysis. Survival curves were estimated using the Kaplan–Meier method and were compared using the log-rank test. The significance was defined as a P value of < 0.05. All statistical analyses were performed using the R2.15.3.

## Results

### Identification and enrichment analysis of MDEGs in GC

**Fig. 1:**
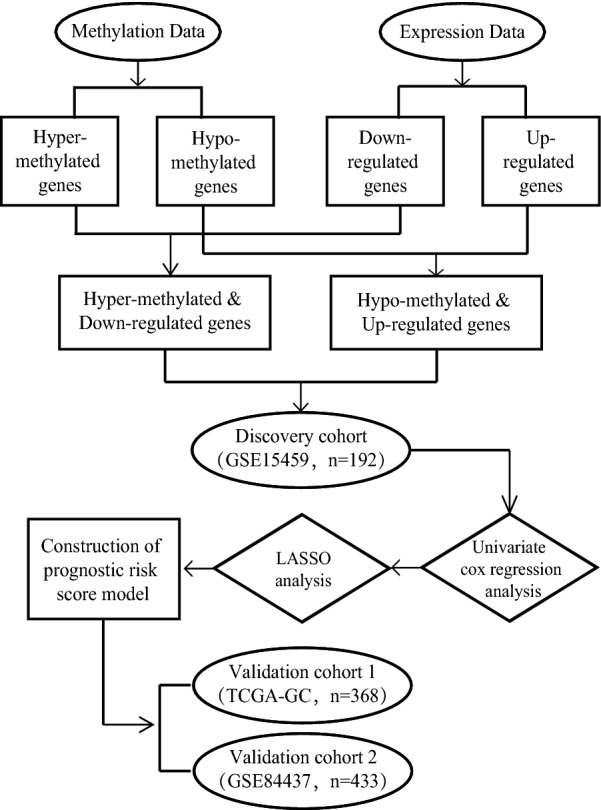
Flowchart of this study

**Fig. 2:**
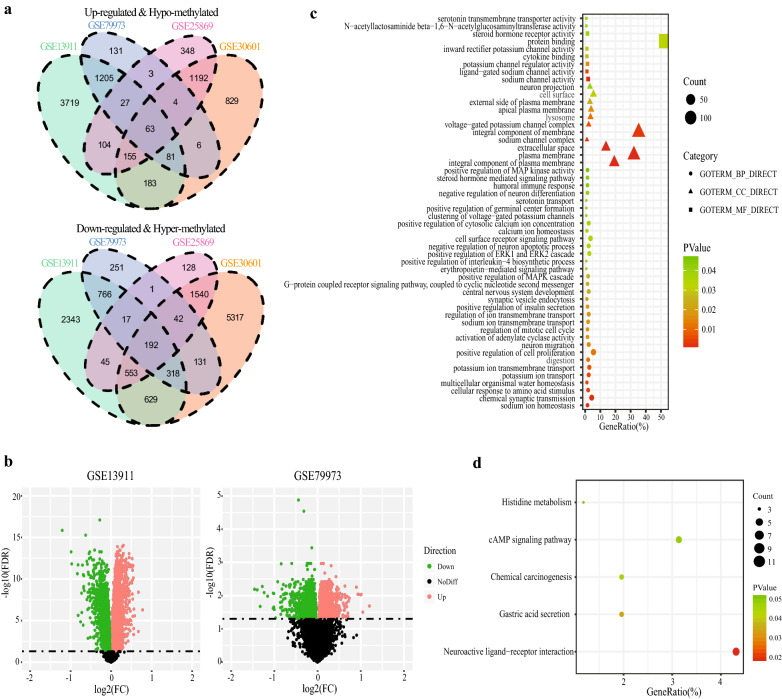
The methylated‐differentially expressed genes identification and function. **a** Venn of methylated‐differentially expressed genes in gene expression datasets (GSE13911, GSE79973) and gene methylation datasets (GSE30601, GSE25869). **b** The volcano plots of GSE13911 and GSE79973 for differentially expressed mRNA. Red and green dots represent significantly up-regulated and down-regulated genes, respectively (FDR < 0.05). **c** The significant enriched gene ontology of MDEGs. **d** The significant enriched KEGG pathways of MDEGs

The flowchart for this study is shown in Fig. 1. With cut-off criteria of FDR < 0.05, 10,400 and 3238 DEGs were identified from GSE13911 and GSE79973 respectively. A total of 2669 DEGs, including 1376 up-regulated genes and 1293 down-regulated genes were concordance. The concordance score was 99.8% (binomial test, P < 0.0001). Similarly, we identified 11,235 and 4414 DMGs from GSE30601 and GSE25869 respectively. A total of 3741 DMGs, including 2327 hyper-methylated genes and 1414 hypo-methylated genes were concordance. The concordance score was 97.7% (binomial test, P < 0.0001). By correlating the level of RNA expression with the degree of DNA methylation, we totally identified 255 MDEGs consisted of 192 hypermethylation-low expression genes and 63 hypomethylation-high expression genes (Fig. 2a, Additional file 1: Table S2). To confirm that the FDR value is logical using a different test, a representative volcano plot was constructed for GSE13911 and GSE79973, respectively (Fig. 2b).

Enrichment analysis with the Database for DAVID was used to elucidate biological function of the MDEGs. The top significant terms emerging from the gene oncology (GO) enrichment analysis are shown in Fig. 2c. MDEGs were enriched in “biological processes of positive regulation of cell proliferation,” “positive regulation MAPK cascade,” “positive regulation ERK1 and ERK2 cascade,” “positive regulation MAP kinase activity,” and “activation of adenylate cyclase acivity.” Regarding molecular function, MDEGs showed enrichment in “cytokine binding” and “protein binding.” Enrichment of cell components was mostly “integral component of plasma membrane,” which suggests MDEGs may play an important role in transcription in GC. Kyoto Encyclopedia of Genes and Genomes (KEGG) analysis suggested that MDEGs were significantly enriched in pathways in “cAMP signaling pathway,” “histidine metabolism,” and “chemical carcinogenesis” (Fig. 2d).

### Construction of the eight-MDEGs prognostic signature for GC

**Fig. 3:**
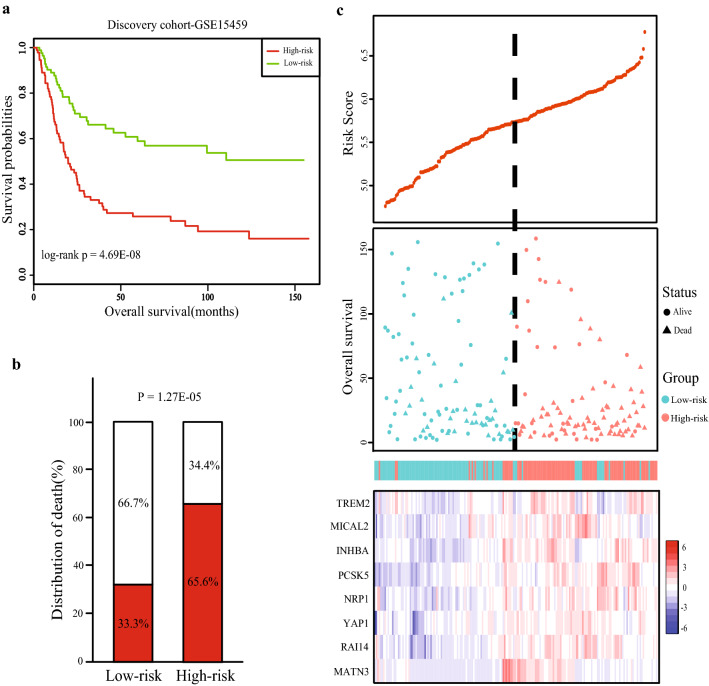
Construction of the eight-MDEGs signature of GC. The patients were stratified into high-risk group and low-risk group based on median of risk score. **a** Kaplan‐Meier curve of the overall survival for high-risk and low-risk scores ranking by the eight-MDEGs signature. **b** The distribution of death in high-risk and low-risk group. **c** Risk score distribution of GC patients, Survival status of each patient and Expression heatmap of the eight MDEGs corresponding to each sample above

Using the univariate Cox regression analysis, we identified MDEGs with potential prognostic value in training cohort. Details of the clinical characteristics are presented in Additional file 1: Table S1. A total of 83 MDEGs including 35 hypomethylation‐high expression genes and 48 hypermethylation‐low expression genes were associated with the overall survival. Based on those prognostic MDEGs, we used the R package “glmnet” to perform Lasso regression analysis. The degree of Lasso regression complexity is controlled by the parameter λ (0 < λ <1). We obtained the optimal value of the parameter λ with the number of variables equal to eight through multiple cross-validation. Therefore, combining the regression coefficients under the optimal λ value, we constructed an eight-MDEGs signature to guide the prognosis of GC patients. The risk-score formula was created as follows: Risk score = (0.185 * expression level of TREM2) + (0.045 * expression level of RAI14) + (0.078 * expression level of NRP1) + (0.043 * expression level of YAP1) + (0.012 * expression level of MATN3) + (0.063 * expression level of PCSK5) + (0.210 * expression level of INHBA) + (0.154 * expression level of MICAL2). We then calculated the risk score for each patient and ranked them based on increasing score, after which patients were classified into a high-risk (n = 96) or a low-risk (n = 96) group based on the median risk score. We observed the overall survival between two risk groups with significantly different survival rate (log-rank P < 0.0001; Fig. 3a). Patients with high risk score had significantly shorter OS than patients with low risk score. OS rates among patients were 34.4% in the high-risk group, as compared to 66.7% in the low-risk group (Fig. 3b). The risk score distribution, survival status, and expression profile of the eight prognostic MDEGs are shown in Fig. 3c. Taking into the patients’ clinical features, including age, gender and stage, the Multivariate Cox regression analysis showed that the eight-MDEGs signature risk score also had statistical significance as an independent prognostic factor in the training cohorts (HR = 2.28, 95% CI 1.47–3.53, P = 2.22E−04) (Table 2).

**Table 2:** Multivariate analysis of prognostic factors by Cox proportional hazard model

Variables	GSE15459		TCGA-GC		GSE84437	
	HR (95%CI)	P value	HR (95% CI)	P value	HR (95% CI)	P value
Age	1.09 (0.71–1.68)	0.709	1.96 (1.09–3.51)	0.025	1.83 (1.38–2.43)	< 0.001
Eight-MDEGs signature						
High-risk vs. low-risk	2.28 (1.47–3.53)	< 0.001	1.84 (1.13–2.99)	0.015	1.43 (1.09–1.88)	0.011
Gender						
Male vs. female	1.07 (0.68–1.68)	0.779	1.25 (0.71–2.22)	0.449	1.31 (0.96–1.77)	0.085
TNM stage						
III + IV vs. I + II	5.99 (3.27–10.99)	< 0.001	2.24 (1.29–3.91)	0.004	–	–

### Prognostic validation of the eight-MDEGs signature

**Fig. 4:**
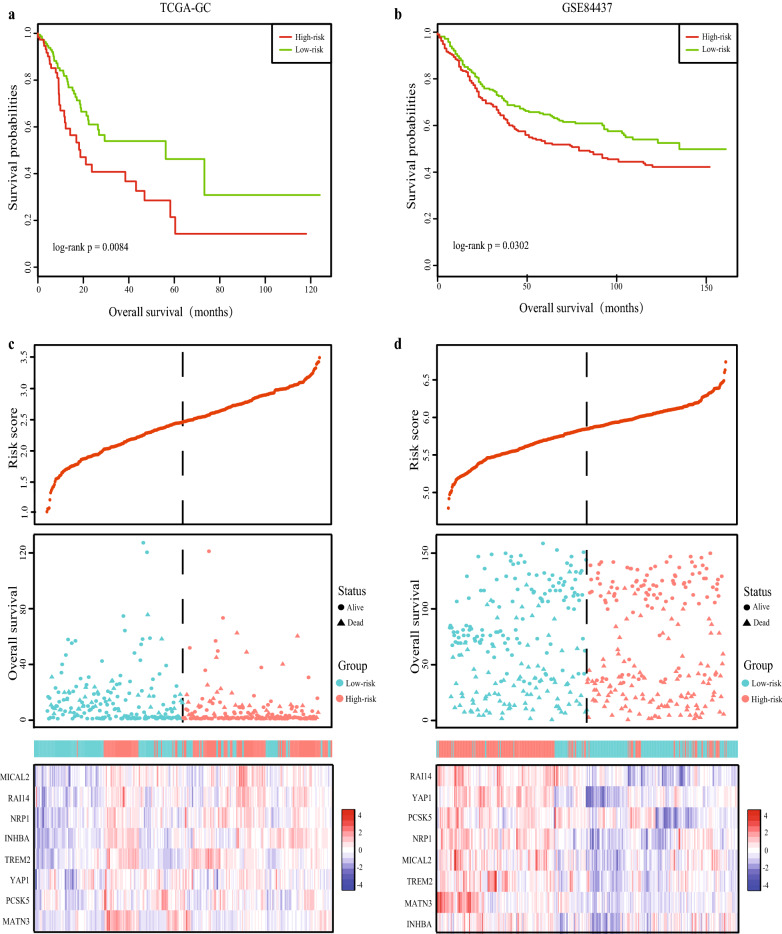
Validation of the eight-MDEGs signature in two independent datasets. Kaplan‐Meier curve of the overall survival for high-risk and low-risk scores ranking by the eight-MDEGs signature in TCGA-GC dataset (**a**) and GSE84437 dataset (**b**). Risk score distribution of GC patients, Survival status of each patient and Expression heatmap of the eight MDEGs corresponding to each sample above in TCGA-GC dataset (**c**) and GSE84437 dataset (**d**)

We validated the prognosis performance of the eight-MDEGs signature in two validation datasets, TCGA-GC and GSE84437 with 368 and 433 patients respectively. Similar to the training cohort findings, patients in high risk group had a shorter survival time than low risk group either in TCGA-GC (HR = 1.85, 95% CI 1.16–2.94, P = 0.0084) or GSE84437 datasets (HR = 1.35, 95% CI 1.03–1.78, P = 0.0302) (Fig. 4a, b). The risk score distribution, survival status, and expression profile of the four prognostic MDEGs are shown in Fig. 4c, d. As missing stage information of partial patients in TCGA, twenty-three patients were excluded when performed multivariate Cox regression analysis. In accordance with the result of the training set, the multivariate Cox regression analysis showed that the eight-MDEGs signature risk score also had statistical significance as an independent variable in the TCGA-GC (HR = 1.84, 95% CI 1.13–2.99, P = 0.015) and GSE84437 (HR = 1.43, 95% CI 1.09–1.88, P = 0.011) respectively (Table 2).

## Discussion

The rapid development of methylation research has provided a novel idea for us to understand the pathogenesis of cancer. Compared to genomic aberrations, DNA‐methylation aberrations are more common in the cancer genome. DNA methylation is the first epigenetic mark shown to be critically involved in the tumorigenesis [15], which provides a stable gene silencing mechanism that plays an important role in regulating gene expression and chromatin architecture. Hypomethylation generally arises early and has been linked to chromosomal instability and loss of imprinting, whereas hypermethylation is associated with promoters and can arise secondary to gene (oncogene suppressor) silencing. The DNA methylation patterns may be potential prognostic indicators of cancer patients and used as a biomarker [16]. Global DNA hypomethylation is mostly seen in GC, even at the early steps of carcinogenesis [17–20]. Li et al. [21] used online bioinformatics resources to explore gastric cancer-specific MDEGs and investigate their potential pathways. Although this study has identified MDEGs in GC, their predictive value for GC patients has not been systematically investigated until now. To our knowledge, this is the first study to develop a MDEG-based risk score that is predictive of prognosis in GC.

In the present study, using methylation and expression microarrays within GEO database, we identified 63 hypomethylation‐high expression genes and 192 hypermethylation‐low expression genes. Enrichment analysis of the MDEGs suggested they were involved in key biological processes, One of key biological processes is sodium ion transmembrane transport, indicating that the channel activity might be affected in GC carcinogenesis [22]. This finding is consistent with the knowledge that ion transport in cancer cells is substantially different from that in normal cells [23]. Another key biological process is G-protein coupled receptor signaling pathway. The roles of G-protein coupled receptor (GPCRs) had been extensively reported related to tumor invasion and metastasis [24–27]. Our finding showed that the abnormal methylation and expression of genes within G-protein coupled receptor signaling pathway played a key role in tumor progression and prognosis. Besides, the defective functioning of the regulation of cell proliferation and MAPK signaling pathway suggest that it really is an important reason for tumor development [28–31]. KEGG enrichment analysis suggested that MDEGs were significantly enrichened in cancer-associated pathways. For example, Wang et al. [32] Found that CREB1 as a member of cAMP signaling pathway was highly expressed and correlated with lymph node metastasis, distant metastasis and tumor stage and poor outcome in gastric cancer. Together, these results suggested that MDEGs played a critical role in gastric cancer development.

Based on LASSO regression analysis, eight MDEGs including TREM2,RAI14, NRP1, YAP1, MATN3, PCSK5, INHBA and MICAL2, were selected for further analysis. Previous studies had reported that almost MDEGs could promote the cell proliferation and contribute to carcinogenesis. Triggering receptor expressed on monocytes 2 (TREM2) is a member of the immunoglobulin superfamily and combines with TYRO protein tyrosine kinase-binding protein to form a complex at the cell Surface. Zhang et al. [33] found that TREM2 expressions were significantly higher in GC compared with normal gastric tissues and were inversely correlated with patient prognosis. Nonetheless, the oncogenic roles and potential molecular mechanisms of TREM2 in gastric cancer remain unknown and need in-depth research. Retinoic acid induced 14 (RAI14), also known as NORPEG, is an actin-binding protein initially observed to be a regulatory protein at the ectoplasmic specialization. Recent studies highlight that RAI14 was up-regulated in gastric cancer associated with the patient’s prognosis and RAI14 knockdown by siRNA interference reduced proliferation and migration, promoted apoptosis through inhibiting the activation of Akt signaling pathway in gastric cancer [34, 35]. Wang et al. [36] proved that NRP1 was a hypomethylated-upregulated gene in GC patients, which was significantly correlated with tumor malignant phenotypes. The expression level of NRP1 was significantly associated with the overall survival of GC patients. Zhang et al. [37] further demonstrated that NRP1 could act as a mediator for iRGD to strengthen the chemotherapy efficacy of 5-FU on gastric cancer cell. Yes-associated protein 1 (YAP1) is a transcriptional effector of the Hippo pathway that regulates intrinsic organ sizes by regulating apoptosis and cell proliferation. Recent studies reported that YAP1 expression was associated with gastric cancer carcinogenesis and malignancy, which suggested that YAP1 could possibly be a potential treatment target for GC [38–40]. Martrilin-3 (MATN3), as a member of von Willebrand factor A domain containing protein family, is thought to be involved in the formation of filamentous networks in the extracellular matrices of various tissues [41]. Zhang et al. [42] found that MATN3 was aberrantly methylated and differentially expressed in gastric cancer and significantly associated with prognosis. As a member of the superfamily of transforming growth factor-β (TGF-β), inhibin βA (INHBA) forms a disulfide-linked homodimer, namely activin A, which strongly induces differentiation of embryonic stem cells. The role that INHBA played in cancers was found to be associated with activin A levels in colorectal cancer [43], prostate cancer [44], and ovarian cancer [45]. It was also discovered that INHBA is involved in cell proliferation in patients with high expressions, and correlated with the tumor-node-metastasis (TNM) stage and venous invasion, making it an independent factor of prognosis after radical gastrectomy for GC [46, 47]. Molecule interacting with CasL (MICAL2), a microtubule associated monooxygenase, is involved in cell growth, axon guidance, vesicle trafficking and apoptosis. Recent studies have demonstrated that MICAL2 is highly expressed in tumor and accelerates tumor progression and it is deemed to be a novel tumor-promoting factor [48, 49]. PCSK5 belongs to the subtilisin-like proprotein convertase family. Reports of the involvement of PCSK5 in cancer are rare. In triple-negative breast cancer (TNBC), a lack of PCSK5 could lead to the bioactivity of growth differentiation factor (GDF11) as a tumor-suppressor [50]. However, the function of PCSK5 in GC is largely unknown. PCSK5 gene presents a high frequency of genomic alterations in GC patients according to cBioportal. The present study indicated the abnormal expression of PSCK5 might play an important role in gastric cancer carcinogenesis and prognosis, which could be a meaningful direction worthy of further exploration.

## Conclusion

Our study identified an independent prognostic signature by combining methylation and expression information, which could successfully classify GC patients into high-risk and low-risk groups with significant differences in OS. The eight-MDEGs signature was promising to be applied for clinical prognostic evaluation of GC patients. These MDEGs may have clinical implications as prognostic markers in GC, which provide information helpful for selection of therapeutic strategies.

## Supplementary information

**Additional file 1: Table S1.** Clinical information analyzed in this study. **Table S2.** List of MDEGs.

## Data Availability

All data and materials are fully available without restriction. The data generated or analyzed during this study are included in this published article.

## Abbreviations

GC: Gastric cancer
GEO: Gene Expression Omnibus
TCGA: The Cancer Genome Atlas
GO: Gene oncology
KEGG: Kyoto Encyclopaedia of Genes and Genomes
LASSO: The least absolute shrinkage and selection operator
OS: Overall survival
CI: Confidence interval
HR: Hazard ratios

## References

[1] Torre LA, Bray F, Siegel RL, Ferlay J, Lortet-Tieulent J, Jemal A (2015). Global cancer statistics, 2012. CA Cancer J Clin.

[2] Torre LA, Siegel RL, Ward EM, Jemal A (2016). Global Cancer Incidence and Mortality Rates and Trends–An Update. Cancer Epidemiol Biomarkers Prev.

[3] Zeng H, Zheng R, Guo Y, Zhang S, Zou X, Wang N, Zhang L, Tang J, Chen J, Wei K (2015). Cancer survival in China, 2003–2005: a population-based study. Int J Cancer.

[4] Ochenduszko S, Puskulluoglu M, Konopka K, Fijorek K, Slowik AJ, Pedziwiatr M, Budzynski A (2017). Clinical effectiveness and toxicity of second-line irinotecan in advanced gastric and gastroesophageal junction adenocarcinoma: a single-center observational study. Ther Adv Med Oncol.

[5] Ushijima T, Asada K (2010). Aberrant DNA methylation in contrast with mutations. Cancer Sci.

[6] Calcagno DQ, Gigek CO, Chen ES, Burbano RR, Smith Mde A (2013). DNA and histone methylation in gastric carcinogenesis. World J Gastroenterol.

[7] Tsai KW, Wu CW, Hu LY, Li SC, Liao YL, Lai CH, Kao HW, Fang WL, Huang KH, Chan WC (2011). Epigenetic regulation of miR-34b and miR-129 expression in gastric cancer. Int J Cancer.

[8] Ziogas D, Roukos D (2009). Epigenetics in gastric cancer: challenges for clinical implications. Ann Surg Oncol.

[9] D’Errico M, de Rinaldis E, Blasi MF, Viti V, Falchetti M, Calcagnile A, Sera F, Saieva C, Ottini L, Palli D (2009). Genome-wide expression profile of sporadic gastric cancers with microsatellite instability. Eur J Cancer.

[10] He J, Jin Y, Chen Y, Yao HB, Xia YJ, Ma YY, Wang W, Shao QS (2016). Downregulation of ALDOB is associated with poor prognosis of patients with gastric cancer. Onco Targets Ther.

[11] Zouridis H, Deng N, Ivanova T, Zhu Y, Wong B, Huang D, Wu YH, Wu Y, Tan IB, Liem N (2012). Methylation subtypes and large-scale epigenetic alterations in gastric cancer. Sci Transl Med.

[12] Kwon OH, Park JL, Kim M, Kim JH, Lee HC, Kim HJ, Noh SM, Song KS, Yoo HS, Paik SG (2011). Aberrant up-regulation of LAMB3 and LAMC2 by promoter demethylation in gastric cancer. Biochem Biophys Res Commun.

[13] Ooi CH, Ivanova T, Wu J, Lee M, Tan IB, Tao J, Ward L, Koo JH, Gopalakrishnan V, Zhu Y (2009). Oncogenic pathway combinations predict clinical prognosis in gastric cancer. PLoS Genet.

[14] Irizarry RA, Hobbs B, Collin F, Beazer-Barclay YD, Antonellis KJ, Scherf U, Speed TP (2003). Exploration, normalization, and summaries of high density oligonucleotide array probe level data. Biostatistics.

[15] Feinberg AP, Vogelstein B (1983). Hypomethylation distinguishes genes of some human cancers from their normal counterparts. Nature.

[16] Lee JJ, Geli J, Larsson C, Wallin G, Karimi M, Zedenius J, Hoog A, Foukakis T (2008). Gene-specific promoter hypermethylation without global hypomethylation in follicular thyroid cancer. Int J Oncol.

[17] Tahara T, Arisawa T (2015). DNA methylation as a molecular biomarker in gastric cancer. Epigenomics.

[18] Choi SJ, Jung SW, Huh S, Chung YS, Cho H, Kang H (2017). Alteration of DNA methylation in gastric cancer with chemotherapy. J Microbiol Biotechnol.

[19] Park JH, Park J, Choi JK, Lyu J, Bae MG, Lee YG, Bae JB, Park DY, Yang HK, Kim TY (2011). Identification of DNA methylation changes associated with human gastric cancer. BMC Med Genomics.

[20] Nakamura J, Tanaka T, Kitajima Y, Noshiro H, Miyazaki K (2014). Methylation-mediated gene silencing as biomarkers of gastric cancer: a review. World J Gastroenterol.

[21] Li H, Liu JW, Liu S, Yuan Y, Sun LP (2017). Bioinformatics-based identification of methylated-differentially expressed genes and related pathways in gastric cancer. Dig Dis Sci.

[22] Djamgoz MB, Coombes RC, Schwab A (2014). Ion transport and cancer: from initiation to metastasis. Philos Trans R Soc Lond B Biol Sci.

[23] Fraser SP, Pardo LA (2008). Ion channels: functional expression and therapeutic potential in cancer. Colloquium on Ion Channels and Cancer. EMBO Rep.

[24] Li S, Huang S, Peng SB (2005). Overexpression of G protein-coupled receptors in cancer cells: involvement in tumor progression. Int J Oncol.

[25] Chatterjee S, Behnam Azad B, Nimmagadda S (2014). The intricate role of CXCR4 in cancer. Adv Cancer Res.

[26] Pan WL, Wang Y, Hao Y, Wong JH, Chan WC, Wan DC, Ng TB (2018). Overexpression of CXCR4 synergizes with LL-37 in the metastasis of breast cancer cells. Biochim Biophys Acta Mol Basis Dis.

[27] Yu X, Wang D, Wang X, Sun S, Zhang Y, Wang S, Miao R, Xu X, Qu X (2019). CXCL12/CXCR4 promotes inflammation-driven colorectal cancer progression through activation of RhoA signaling by sponging miR-133a-3p. J Exp Clin Cancer Res.

[28] Evan GI, Vousden KH (2001). Proliferation, cell cycle and apoptosis in cancer. Nature.

[29] Guo X, Ma N, Wang J, Song J, Bu X, Cheng Y, Sun K, Xiong H, Jiang G, Zhang B (2008). Increased p38-MAPK is responsible for chemotherapy resistance in human gastric cancer cells. BMC Cancer.

[30] Husain SS, Szabo IL, Pai R, Soreghan B, Jones MK, Tarnawski AS (2001). MAPK (ERK2) kinase–a key target for NSAIDs-induced inhibition of gastric cancer cell proliferation and growth. Life Sci.

[31] Wagner EF, Nebreda AR (2009). Signal integration by JNK and p38 MAPK pathways in cancer development. Nat Rev Cancer.

[32] Wang YW, Chen X, Gao JW, Zhang H, Ma RR, Gao ZH, Gao P (2015). High expression of cAMP-responsive element-binding protein 1 (CREB1) is associated with metastasis, tumor stage and poor outcome in gastric cancer. Oncotarget.

[33] Zhang X, Wang W, Li P, Wang X, Ni K (2018). High TREM2 expression correlates with poor prognosis in gastric cancer. Hum Pathol.

[34] He XY, Zhao J, Chen ZQ, Jin R, Liu CY (2018). High expression of retinoic acid induced 14 (RAI14) in gastric cancer and its prognostic value. Med Sci Monit.

[35] Chen C, Maimaiti A, Zhang X, Qu H, Sun Q, He Q, Yu W (2018). Knockdown of RAI14 suppresses the progression of gastric cancer. Onco Targets Ther.

[36] Wang G, Shi B, Fu Y, Zhao S, Qu K, Guo Q, Li K, She J (2019). Hypomethylated gene NRP1 is co-expressed with PDGFRB and associated with poor overall survival in gastric cancer patients. Biomed Pharmacother.

[37] Zhang L, Xing Y, Gao Q, Sun X, Zhang D, Cao G (2017). Combination of NRP1-mediated iRGD with 5-fluorouracil suppresses proliferation, migration and invasion of gastric cancer cells. Biomed Pharmacother.

[38] Yu L, Gao C, Feng B, Wang L, Tian X, Wang H, Ma D (2017). Distinct prognostic values of YAP1 in gastric cancer. Tumour Biol.

[39] Du F, Yu C, Li R, Ding D, He L, Wen G (2019). Expression of miR-141 and YAP1 in gastric carcinoma and modulation of cancer cell proliferation and apoptosis. Int J Clin Exp Pathol.

[40] Sun D, Li X, He Y, Li W, Wang Y, Wang H, Jiang S, Xin Y (2016). YAP1 enhances cell proliferation, migration, and invasion of gastric cancer in vitro and in vivo. Oncotarget.

[41] Wu PL, He YF, Yao HH, Hu B (2018). Martrilin-3 (MATN3) overexpression in gastric adenocarcinoma and its prognostic significance. Med Sci Monit.

[42] Zhang C, Liang Y, Ma MH, Wu KZ, Dai DQ (2019). KRT15, INHBA, MATN3, and AGT are aberrantly methylated and differentially expressed in gastric cancer and associated with prognosis. Pathol Res Pract.

[43] Okano M, Yamamoto H, Ohkuma H, Kano Y, Kim H, Nishikawa S, Konno M, Kawamoto K, Haraguchi N, Takemasa I (2013). Significance of INHBA expression in human colorectal cancer. Oncol Rep.

[44] Hofland J, van Weerden WM, Steenbergen J, Dits NF, Jenster G, de Jong FH (2012). Activin A stimulates AKR1C3 expression and growth in human prostate cancer. Endocrinology.

[45] Dean M, Davis DA, Burdette JE (2017). Activin A stimulates migration of the fallopian tube epithelium, an origin of high-grade serous ovarian cancer, through non-canonical signaling. Cancer Lett.

[46] Oshima T, Yoshihara K, Aoyama T, Hasegawa S, Sato T, Yamamoto N, Akito N, Shiozawa M, Yoshikawa T, Numata K (2014). Relation of INHBA gene expression to outcomes in gastric cancer after curative surgery. Anticancer Res.

[47] Chen ZL, Qin L, Peng XB, Hu Y, Liu B (2019). INHBA gene silencing inhibits gastric cancer cell migration and invasion by impeding activation of the TGF-beta signaling pathway. J Cell Physiol.

[48] Mariotti S, Barravecchia I, Vindigni C, Pucci A, Balsamo M, Libro R, Senchenko V, Dmitriev A, Jacchetti E, Cecchini M (2016). MICAL2 is a novel human cancer gene controlling mesenchymal to epithelial transition involved in cancer growth and invasion. Oncotarget.

[49] Cai Y, Lu J, Tang F (2018). Overexpression of MICAL2, a novel tumor-promoting factor, accelerates tumor progression through regulating cell proliferation and EMT. J Cancer.

[50] Bajikar SS, Wang CC, Borten MA, Pereira EJ, Atkins KA, Janes KA (2017). Tumor-suppressor inactivation of GDF11 occurs by precursor sequestration in triple-negative breast cancer. Dev Cell.

