# Supplementary material for: A methylation‐based mRNA signature predicts survival in patients with gastric cancer
Source: Cancer Cell Int. 2020 Jul 6;20:284. doi: 10.1186/s12935-020-01374-w (PMC7336496; doi:10.1186/s12935-020-01374-w)
Supplement: Supplementary file 1 — Additional file 1: Table S1. Clinical information analyzed in this study. Table S2. List of MDEGs. [file 12935_2020_1374_MOESM1_ESM.docx]

**Table S1 Clinical information analyzed in this study**

|  | **Training dataset** | **Validation**  **dataset** | |
| --- | --- | --- | --- |
|  | **GSE15459** | **TCGA-GC** | **GSE84437** |
| **Sample** |  |  |  |
| Normal | - | - | - |
| Tumor | 192 | 368 | 433 |
| **Mean age**  (years;range) | 64  (23-92) | 66  (35-90) | 60  (27-86) |
| **Gender** |  |  |  |
| Male | 125 | 236 | 296 |
| Female | 67 | 132 | 137 |
| **Stage** |  |  |  |
| I | 31 | 49 | - |
| II | 29 | 110 | - |
| III | 72 | 149 | - |
| IV | 60 | 37 | - |
| Unknown | 0 | 23 | - |
| **Status** |  |  |  |
| Alive | 97 | 295 | 224 |
| Dead | 95 | 73 | 209 |
| **Platform** | Affymetrix U133 Plus 2 | Illumina HiSeqV2 | Illumina HT-12 V3 |

**Table S2 List of MDEGs**

|  | **Gene list** |
| --- | --- |
| **Hypermethylation-low expression genes** | PGC;RGS5;KCNE2;KCNJ13;FBP2;RILP;MFSD7;ESR1;RORC;FIGF;GPR25;IRF4;KCNB2;GRASP;IGFALS;BMP6;VSIG1;SIK1;CIDEB;PPAP2B;NEURL;CAMK2B;CXCL17;PDGFD;FMO4;SSTR2;BOLL;CAPN2;ADHFE1;ANO7;PNLIPRP2;NODAL;MYRIP;SPINK2;CPEB4;ATP8B1;COL23A1;C3orf57;CCDC105;GCNT2;ADAMTSL1;NGEF;SHROOM3;SLC1A2;NRSN2;GCNT4;SSBP3;ACSS1;DISP1;NPY2R;MAL;ATP5F1;ADAMTS13;KLK11;APOBEC2;STON2;C4orf33;KCNIP2;ASCL1;IGF2AS;SCNN1A;EPOR;RPRM;C20orf151;SOX2;SPATA18;DNAJC4;ATP8A2;CACNG2;GPT;CYP3A43;SELENBP1;VSIG2;ASTN1;SSTR4;OPCML;C11orf9;GAMT;UPK1B;PLD5;HAPLN1;ALDH3A1;KCNMB2;ADRB2;EPHA8;RNF152;CIDEA;LMX1A;KLHDC7A;FAM20A;RFX6;HMGCS2;ACSF2;SCNN1B;ENTPD5;CWH43;RBM47;GIPR;BLNK;HHIP;C6orf105;HS6ST3;NKX3-1;CAPN9;MOGAT2;RDH12;KCNH7;TFAP2B;VGLL2;TRPM3;CCDC60;EYA2;CLCNKB;BHMT;SCGB2A1;HPCA;ADA;TNFRSF13C;C15orf27;FLJ45983;CLIC6;PGLYRP1;DCAF12L1;OSBPL7;SEZ6L;SLC18A2;CNTN2;HTR6;VSTM2A;SLC18A1;OTOP2;PGPEP1;DHRS12;GABBR2;D4S234E;SCNN1G;ACCN4;RANBP3L;INSRR;SIM1;NCR1;SH3GL2;NTN4;POU2F2;ASPA;SEPT12;ADH1C;CSMD1;NPPB;DAPK2;GCKR;C9orf95;KLF15;LTF;EEF1A2;CADM3;PACSIN1;ZNF323;SPATA4;HDC;COX6A2;METTL7A;KCNH3;C11orf52;CNTFR;CCKBR;GPX3;BAIAP3;SLC28A1;PEX11G;C13orf36;TMCC2;C7orf52;KCNJ16;C8A;ESRRG;TMEM175;CYP3A5;SULT1B1;IL5RA;ATRN;DRD5;NR0B2;FRMD1;DNASE1L2;KAAG1;HPCAL4;MPPED1;HRH3;MT3;SFRP5;KIT |
| **Hypomethylation-high expression genes** | HAVCR2;C3AR1;FCGR2A;IPO5;THOC6;MNDA;ROBO1;FAP;MSR1;TEAD4;UACA;PI15;NACC2;CLEC4A;LCA5;C3orf39;LAPTM5;ITGB1BP2;TREM2;H19;CCDC93;GJA1;JUB;CDC42BPB;C1orf59;RAI14;NDUFAF2;ASAP1;NRP1;IGSF6;RPA3;GNB5;TYROBP;YAP1;POP1;TMEM43;ZNF703;CAP2;HSD11B1;TNFSF11;TMEM173;S100A10;ACTN1;FCER1G;CD86;MATN3;NINJ2;SLAMF8;PCSK5;INHBA;MORF4L1;CCT6A;UBE2V2;LAMB1;COL6A3;ENTPD1;LBR;S100A4;HIVEP2;AFAP1;MICAL2;FKBP9;SLC4A11 |
